# Clinical Analysis of *Acinetobacter* Species Infections in Children and Adolescents Treated for Cancer or Undergoing Hematopoietic Cell Transplantation: A Multicenter Nationwide Study

**DOI:** 10.3390/jcm14144928

**Published:** 2025-07-11

**Authors:** Ewelina Truszkowska, Krzysztof Czyżewski, Katarzyna Derwich, Kamila Jaremek, Oliwia Grochowska, Patrycja Zalas-Więcek, Katarzyna Pawińska-Wąsikowska, Wojciech Czogała, Szymon Skoczeń, Walentyna Balwierz, Małgorzata Salamonowicz-Bodzioch, Krzysztof Kałwak, Aleksandra Królak, Tomasz Ociepa, Tomasz Urasiński, Filip Pierlejewski, Małgorzata Nowak, Maciej Zdunek, Wojciech Młynarski, Olga Gryniewicz-Kwiatkowska, Magdalena Łukszo, Bożenna Dembowska-Bagińska, Anna Szmydki-Baran, Łukasz Hutnik, Aleksandra Minkowska, Katarzyna Pikora, Paweł Łaguna, Marcin Płonowski, Maryna Krawczuk-Rybak, Tomasz Brzeski, Katarzyna Mycko, Wanda Badowska, Weronika Stolpa, Karolina Baranowska, Agnieszka Mizia-Malarz, Ewa Bień, Ninela Irga-Jaworska, Renata Tomaszewska, Agnieszka Książek, Tomasz Szczepański, Wioletta Bal, Radosław Chaber, Agnieszka Urbanek-Dądela, Grażyna Karolczyk, Sonia Pająk, Stefania Krawczyk, Katarzyna Machnik, Jan Styczyński, Olga Zając-Spychała

**Affiliations:** 1 Department of Pediatric Oncology, Hematology and Transplantology, University of Medical Sciences, Szpitalna 27/33, 60-572 Poznan, Poland; 2 Department of Pediatric Hematology and Oncology, Collegium Medicum in Bydgoszcz, Nicolaus Copernicus University Torun, Marii Skłodowskiej-Curie 9, 85-094 Bydgoszcz, Poland; 3 Department of Microbiology, Collegium Medicum, Nicolaus Copernicus University Torun, Marii Skłodowskiej-Curie 9, 85-094 Bydgoszcz, Poland; 4 Department of Pediatric Oncology and Hematology, Institute of Pediatrics, Jagiellonian University Medical College, Wielicka 265, 30-663 Krakow, Poland; 5 Department of Pediatric Stem Cell Transplantation, Hematology and Oncology, Medical University, Borowska 213, 50-556 Wroclaw, Poland; 6 Department of Pediatrics, Hemato-Oncology and Gastroenterology, Pomeranian Medical University, Unii Lubelskiej 1, 71-252 Szczecin, Poland; 7 Department of Pediatrics, Oncology and Hematology, Medical University, Sporna 36/50, 91-738 Lodz, Poland; 8 Department of Oncology, Children’s Memorial Health Institute, Dzieci Polskich 20, 04-730 Warsaw, Poland; 9 Department of Oncology, Pediatric Hematology, Clinical Transplantology and Pediatrics, Medical University, Żwirki i Wigury 63A, 02-091 Warszawa, Poland; 10 Department of Pediatric Oncology and Hematology, Medical University, Waszyngtona 17, 15-274 Bialystok, Poland; 11 Clinical Divison of Pediatric Oncology and Hematology, University of Warmia and Mazury, Regional Specialised Children’s Hospital, Żołnierska 18a, 10-561 Olsztyn, Poland; 12 Department of Pediatric Hematology and Oncology, Medical University of Silesia, Medykow 16, 40–752 Katowice, Poland; 13 Department of Pediatrics, Hematology and Oncology, Medical University, Dębinki 7, 80-211 Gdansk, Poland; 14 Department of Pediatric Hematology and Oncology, Medical University of Silesia, 3-go Maja 13-15, 41-800 Zabrze, Poland; 15 Department of Pediatric Oncohematology, Clinical Provincial Hospital No. 2, Lwowska 60, 35-301 Rzeszow, Poland; 16 Division of Pediatric Hematology and Oncology, Regional Polyclinic Hospital, Artwińskiego 3a, 25-734 Kielce, Poland; 17 Division of Pediatric Hematology and Oncology, Chorzow City Hospital, Władysława Truchana 7, 41-500 Chorzow, Poland

**Keywords:** *Acinetobacter*, pediatric oncology, antibiotic resistance

## Abstract

**Background**: *Acinetobacter*, specifically *A. baumannii*, are becoming a great threat to hospitalized patients due to increasing antibiotic resistance. The aim of this study was to describe the epidemiology, clinical characteristics, antimicrobial susceptibility pattern and outcome of *Acinetobacter* infections in pediatric cancer patients and hematopoietic stem cell transplant (HSCT) recipients in Poland. **Methods**: A total of 125 episodes of *Acinetobacter* species infections were reported in patients <18 years treated in Polish pediatric hematology and oncology centers over a period from 2012 to 2023. Infections were subdivided into oncohematological disease (OHD) group (*n* = 106; 84.8%) and HSCT group (*n* = 19; 15.2%). Each episode represented a separate infection event; therefore, a patient who was infected more than once during the course of treatment was counted for each infection episode. **Results**: *A. baumannii* is the most common *Acinetobacter* species in all groups. The most common diagnoses in OHD group were acute lymphoblastic leukemia (ALL) (*n* = 32; 30.2%) and acute myeloid leukemia (AML) (*n* = 13; 12.3%). The most common underlying diseases that were indication for HSCT were hemophagocytic lymphohistiocytosis (*n* = 3; 15.8%) and neuroblastoma (*n* = 3; 15.8%). Mortality was significantly higher in the HSCT group compared to the OHD group. In the OHD group, deaths did not correlate with the type of antibiotic, with an exception for gentamicin, which correlated with higher mortality. In the HSCT group, deaths did not correlate with the type of antibiotic, except for levofloxacin that was correlated with a higher mortality rate. **Conclusions**: *Acinetobacter* infections are a great danger to immunocompromised patients. More research is needed in order to prevent and treat antibiotic-resistant bacteria.

## 1. Introduction

The *Acinetobacter* genus includes a diverse group of glucose non-fermenting, catalase-positive, oxidase-negative, aerobic Gram-negative coccobacilli. Among them, *Acinetobacter baumannii* is the most clinically significant species [1]. It is commonly associated with healthcare-associated infections, particularly in intensive care unit (ICU) patients. The infections it causes include ventilator-associated pneumonia, bloodstream infections related to central venous catheters, urinary tract infections (especially in patients with catheters or nephrostomy tubes), wound infections, and meningitis [2].

*A. baumannii* is known for its remarkable ability to acquire resistance to multiple antibiotics, including carbapenems, making it a serious global public health threat. Recognizing its clinical importance, the World Health Organization (WHO) has listed *A. baumannii* as one of the ESKAPE pathogens: a group of six highly virulent and antibiotic-resistant bacteria that pose significant treatment challenges. This acronym stands for *Enterococcus faecium, Staphylococcus aureus, Klebsiella pneumoniae, Acinetobacter baumannii, Pseudomonas aeruginosa*, and *Enterobacter* species [1].

The resistance mechanisms of *A. baumannii* are diverse. It shows intrinsic resistance to several antibiotics, including aminopenicillins, trimethoprim, ertapenem, tetracycline, aztreonam [3], and fosfomycin [4]. It produces a variety of beta-lactamases, such as the *Acinetobacter*-derived cephalosporinase (ADC), a type of AmpC beta-lactamase, and extended-spectrum beta-lactamases (ESBLs), which confer resistance to third- and fourth-generation cephalosporins [5]. Carbapenem resistance is primarily mediated by class D OXA-type carbapenemases and decreased membrane permeability [6]. Other resistance mechanisms include aminoglycoside-modifying enzymes and active efflux pumps [7].

In addition to *A. baumannii*, other *Acinetobacter* species, often referred to as non-baumannii species—most notably *A. pittii* and *A. nosocomialis*—are increasingly being recognized as clinically relevant pathogens [8]. However, data on their clinical significance, microbiological characteristics, and antibiotic resistance profiles remain limited, particularly in pediatric populations.

Children and adolescents with cancer or undergoing hematopoietic stem cell transplantation (HSCT) are particularly vulnerable to severe infections. In Poland, approximately 1200 new cases of pediatric cancer are diagnosed annually, and a total of 179 hematopoietic stem cell transplantations were performed in pediatric patients in Poland in 2021 [9]. These children often require prolonged hospitalizations, intensive antimicrobial therapy, central venous access, and immunosuppressive treatment, all of which increase their risk of healthcare-associated infections by multidrug-resistant organisms such as *Acinetobacter* species. Despite this, data on the epidemiology and outcomes of *Acinetobacter* infections in this population remain scarce.

The aim of this multicenter, nationwide study was to describe the epidemiology, clinical characteristics, antimicrobial susceptibility patterns, and outcomes of *Acinetobacter* infections in pediatric cancer patients and hematopoietic stem cell transplant (HSCT) recipients treated at Polish pediatric hematology and oncology (PHO) centers and HSCT centers over a 12-year period (2012–2023).

## 2. Materials and Methods

### 2.1. Design of the Study

This was a retrospective study. Given the nature of the study, the requirement to obtain informed consent from each patient was waived. Medical records were submitted by each Polish pediatric oncology and transplant center, and the data were analyzed centrally. The study was approved by the local Ethics Committee.

### 2.2. Patients

Between 2012 and 2023, a total of 125 episodes of *Acinetobacter* species infections were identified in patients aged 0 to 18 years who were treated at Polish pediatric hematology and oncology centers. All microbiologically confirmed *Acinetobacter* infection episodes in patients who were diagnosed with an oncohematological disease and/or had undergone HSCT during the study period in this age group were included in this retrospective, multicenter, nationwide study. There were no other exclusion criteria. Each episode represents a separate infection event; therefore, a patient who was infected more than once during the course of treatment was counted for each infection episode. The infections were categorized into two groups: the oncohematological disease (OHD) group (*n* = 106; 84.8%) and the HSCT group (*n* = 19; 15.2%). The characteristics of analyzed groups are given in Table 1 and Table 2.

All patients received treatment in accordance with the treatment regimens currently in use at the time of their diagnosis. All hematopoietic stem cell transplantations were performed following institutional procedures and treatment protocols. A uniform standard of antimicrobial prophylaxis was applied to all patients, consisting of trimethoprim/sulfamethoxazole administered three times weekly as prophylaxis against *Pneumocystis jirovecii*, along with non-pharmacological measures. These included strict hand hygiene before contact with the patient and the use of maximal sterile barrier precautions during central venous catheter placement. In all transplanted patients, routine antibacterial prophylaxis was also implemented during the neutropenic phase and immunosuppressive treatment. This prophylaxis included the administration of penicillin, cephalosporins, or ciprofloxacin.

### 2.3. Statistical Analysis

All statistical analyses were conducted using PQStat software (version 1.8.6.122, PQStat Software, Poznań, Poland, 2024). The distribution of continuous variables was assessed using the Shapiro–Wilk test. Descriptive statistics were presented as percentages for categorical variables, as mean values with standard deviations (SD) for normally distributed continuous variables, or as median values with interquartile ranges (IQR) for variables that did not follow a normal distribution. The prevalence of categorical variables was compared using the chi-squared test or the chi-squared test with Yates’ correction, as appropriate. Differences between the two study groups were analyzed using the Mann–Whitney U test. A *p*-value of less than 0.05 was considered statistically significant.

## 3. Results

### 3.1. Demographics and Incidence

During the study period, a total of 106 *Acinetobacter* infections were recorded in the subgroup of patients with oncohematological diseases (OHDs) without hematopoietic stem cell transplantation (HSCT), including 41 girls and 65 boys. The median age was 5.62 years (range: 2.42–13.57 years). The most common diagnoses among infected patients were acute lymphoblastic leukemia (ALL) (*n* = 32; 30.2%), acute myeloid leukemia (AML) (*n* = 13; 12.3%), and central nervous system (CNS) tumors (*n* = 8; 7.5%). In the HSCT subgroup, 19 *Acinetobacter* spp. infections were observed in 9 girls and 10 boys, with a median age of 1.69 years (range: 0.84–9.52 years). The most frequent underlying conditions that served as indications for HSCT among infected patients were hemophagocytic lymphohistiocytosis (*n* = 3; 15.8%) and neuroblastoma (*n* = 3; 15.8%).

### 3.2. Clinical and Microbiological Characteristics

Among the 106 episodes of *Acinetobacter* infections in the OHD group, the most common were bloodstream infections (BSIs; *n* = 69; 65.1%), followed by soft tissue infections (STIs; *n* = 6; 5.7%), respiratory tract infections (RTIs; *n* = 5; 4.7%), and urinary tract infections (UTIs; *n* = 4; 3.8%). The most frequently identified species were *A. baumannii* (*n* = 54; 51.0%), *A. lwoffii* (*n* = 18; 17.0%), and *A. ursingii* (*n* = 9; 8.5%). Additionally, *A. junii* was identified in 7 cases (6.6%), and *A. pittii* in 6 cases (5.7%). In the HSCT subgroup, among 19 episodes of *Acinetobacter* infections, bloodstream infections were the most frequent (*n* = 13; 68.4%), followed by central venous catheter-related infections (*n* = 3; 15.8%). The predominant species identified were *A. baumannii* (*n* = 13; 68.4%) and *A. lwoffii* (*n* = 2; 10.5%), followed by *A. johnsonii* (*n* = 1; 5.3%), *A. lactucae* (*n* = 1; 5.3%), and *A. dijkshoorniae* (*n* = 1; 5.3%).

Microbiological and clinical characteristics of *Acinetobacter* infections in both groups are summarized in Table 3.

There was no significant correlation between the age at the time of the infectious episode and transplant status (*p* = 0.105), nor between blood culture results and transplant status (*p* = 0.444). Additionally, age did not correlate with blood culture positivity (*p* = 0.488).

### 3.3. Antimicrobial Susceptibility and Resistance

In the OHD group, *A. baumannii* strains were most often susceptible to amikacin (*n* = 20; 37.0%). Other susceptibilities included gentamicin (*n* = 17; 31.5%), imipenem (*n* = 17; 31.5%), meropenem (*n* = 15; 27.8%), and trimethoprim/sulfamethoxazole (*n* = 14; 25.9%).

*A. lwoffii* strains were susceptible to meropenem (*n* = 11; 61.1%), amikacin (*n* = 11; 61.1%), imipenem (*n* = 8; 44.4%), gentamicin (*n* = 7; 38.9%), and trimethoprim/sulfamethoxazole (*n* = 5; 27.8%). *A. junii* strains were susceptible to meropenem (*n* = 4; 57.1%), amikacin (*n* = 4; 57.1%), ciprofloxacin (*n* = 3; 42.9%), and ceftazidime (*n* = 3; 42.9%). *A. ursingii* strains were susceptible to meropenem (*n* = 5; 55.6%), imipenem (*n* = 4; 44.4%), trimethoprim/sulfamethoxazole (*n* = 3; 33.3%), ciprofloxacin (*n* = 3; 33.3%), and amikacin (*n* = 3; 33.3%). *A. pittii* strains were susceptible to imipenem (*n* = 3; 50.0%), meropenem (*n* = 3; 50.0%), amikacin (*n* = 3; 50.0%), and trimethoprim/sulfamethoxazole (*n* = 3; 50.0%). *A. baumannii* strains most frequently exhibited resistance to ciprofloxacin (*n* = 11; 20.4%), followed by trimethoprim/sulfamethoxazole (*n* = 8; 14.8%), amikacin (*n* = 7; 13.0%), and piperacillin/tazobactam (*n* = 7; 13.0%). Only three strains (5.6%) were classified as multidrug-resistant (MDR).

In the HSCT group, the identified *A. baumannii* strains were most often multisusceptible (*n* = 5; 38.5%), followed by susceptibility to amikacin (*n* = 3; 23.1%) and colistin (*n* = 3; 23.1%). *A. johnsonii* was susceptible to trimethoprim/sulfamethoxazole (*n* = 1; 7.7%). *A. dijkshoorniae* showed susceptibility to amikacin (*n* = 1; 7.7%), levofloxacin (*n* = 1; 7.7%), tobramycin (*n* = 1; 7.7%), and trimethoprim/sulfamethoxazole (*n* = 1; 7.7%). *A. lwoffii* and *A. lactucae* strains were multisusceptible (*n* = 2 and *n* = 1, respectively). *A. baumannii* strains in this group were most often resistant to carbapenems (*n* = 3; 23.1%). One strain (7.7%) was classified as MDR. *A. johnsonii* was resistant to carbapenems and aminoglycosides, specifically tobramycin (*n* = 1; 7.7% each). *A. dijkshoorniae* was resistant to imipenem and meropenem (*n* = 1; 7.7% each).

### 3.4. Antibiotic Therapy Applied

In the OHD group, monotherapy was used in 16 cases of *A. baumannii* infections, accounting for 29.6% of the cases. Combination therapy with two antibiotics was used in 15 episodes (27.8%), while therapy with three antibiotics was applied in 12 episodes (22.2%). In addition, four-antibiotic regimens were used in 3 cases (5.6%), and five-antibiotic regimens in 2 cases (3.7%). Meropenem and amikacin were the most frequently used antibiotics in treating *Acinetobacter* infections. For *A. baumannii*, meropenem was used in 18 cases (33.3%), amikacin in 14 cases (25.9%), and piperacillin/tazobactam in 8 cases (14.8%). In infections caused by *A. lwoffii*, meropenem was administered in 8 cases (44.4%) and amikacin in 7 cases (38.9%). In infections where the species was not identified, meropenem was used in 3 cases (50.0%) and cefepime in 2 cases (33.3%). For *A. ursingii* infections, amikacin was used in 6 cases (66.7%) and meropenem in 4 cases (44.4%). All known *A. pittii* infections were treated with amikacin, although, in two cases, the details of treatment were unavailable.

In the HSCT group, monotherapy was used in two cases of *A. baumannii* infection (15.4%). Combination therapy with two antibiotics was applied in five episodes (38.5%), with three antibiotics in three episodes (23.1%), and with five antibiotics in one episode (7.7%). The most commonly used antibiotics for *A. baumannii* infections were amikacin (six cases; 46.2%), meropenem (five cases; 38.5%), colistin (four cases; 30.8%), and teicoplanin (three cases; 23.1%). Infections caused by *A. lwoffii* were treated with meropenem in both cases (100%), and either cefepime or colistin in one case each (50.0%). *A. johnsonii* infection was treated with meropenem, *A. lactucae* with either piperacillin/tazobactam or meropenem, and the single case of *A. dijkshoorniae* infection was treated with amikacin.

### 3.5. Treatment Outcomes

In the OHD group, a total of 10 deaths were reported. None of these were directly attributed to *Acinetobacter* spp. infection. The primary cause of death was cancer progression, which accounted for 6 cases (60.0%). Other causes included infections in 2 cases (20.0%), treatment-related complications in 1 case (10.0%), and unknown cause in 1 case (10.0%). There was no significant correlation between mortality and the type of antibiotic used, with the exception of gentamicin, where use was associated with a higher mortality rate (*p* = 0.021).

In the HSCT group, five deaths were reported. One of these was attributed directly to *A. baumannii* infection. The leading causes of death were transplant-related complications, which accounted for three cases (60.0%), including two cases of veno-occlusive disease (40.0%) and one case of Epstein–Barr virus-associated post-transplant lymphoproliferative disorder (20.0%). Cancer progression was the cause of death in one case (20.0%). In this group, mortality did not significantly correlate with gender (*p* = 0.089) or age (*p* = 0.754). Additionally, the type of antibiotic therapy was not associated with increased mortality, except for levofloxacin, which was linked to a higher mortality rate (*p* = 0.046). Overall, mortality was significantly higher in the HSCT group compared to the OHD group (*p* = 0.043).

## 4. Discussion

*A. baumanii* being the most common *Acinetobacter* species belongs to the ESKAPE group due to its rising antibiotic resistance, high prevalence, and mortality. It poses a major threat to public health, causing outbreaks in hospital departments [1]. *Acinetobacter* spp. infections became an issue for pediatric cancer patients as well, who are particularly at risk due to severe immunosuppression caused by cancer itself or treatment and lengthy stays at the hospital.

Two Egyptian studies have provided insight into the genetic mechanisms of resistance in *A. baumannii* strains isolated from pediatric cancer patients [10,11]. Al-Hassan et al. [10] identified a broad range of blaOXA-51-like genes and acquired class D carbapenemases (OXA-23, OXA-40, and OXA-58), contributing to extensive carbapenem resistance. Jalal et al. [11], using whole-genome sequencing of 31 MDR isolates, revealed the presence of diverse plasmid lineages and genes encoding multiple resistance mechanisms, including β-lactamases, efflux pumps, and insertion sequences (ISAba1, ISAba125) that enhance β-lactamase expression. Mutations in outer membrane proteins were also reported, potentially contributing to colistin resistance.

A study conducted in Brazil by Costa et al. [12] examined Gram-negative bacterial (GNB) infections in pediatric oncology intensive care units of tertiary oncology public hospitals. Nearly 50% of all infectious episodes were caused by MDR GNB, with *A. baumannii* being the most frequently identified MDR pathogen. Among non-MDR GNB, *A. baumannii* was the second most common isolate. Unlike our study, Costa et al. [12] reported that bacteria were most often isolated from tracheal aspirates, followed by blood and urine cultures. This may be attributed to the higher use of invasive procedures such as mechanical ventilation in ICU settings. The most common underlying malignancies in their cohort were central nervous system tumors and neuroblastoma, whereas in our cohort, ALL was most frequent, followed by AML and CNS tumors. Notably, only one patient in the Brazilian study had undergone HSCT. Their findings also highlight the importance of timely and adequate empirical antibiotic therapy. Patients with MDR GNB infections were more likely to receive inappropriate initial treatment, and the delay in initiating effective therapy was longer in this group. Although the difference was not statistically significant, 30-day mortality was higher in the MDR GNB group (25.5%) compared to the non-MDR GNB group (16.7%) [12].

Further evidence on pediatric MDR *A. baumannii* infections comes from Shi et al. [13], who investigated cases in a pediatric ICU. Similarly to Costa et al. [12], most isolates were obtained from the lower respiratory tract, with ventilator-associated pneumonia being the most frequent complication. The mortality rate associated with *A. baumannii* infections in their study was 16.7%, with bloodstream infections and meningitis also commonly observed. Interestingly, patients in this cohort exhibited reduced natural killer (NK) cell activity, an increased CD4+ T-cell ratio, and elevated serum interleukin-8 levels. Higher serum creatinine and a high blood urea nitrogen-to-albumin ratio were associated with increased mortality risk, suggesting potential roles for these parameters as prognostic biomarkers. Resistance to β-lactams exceeded 75% in this study, underscoring the therapeutic challenge posed by these infections [13].

Zhu et al. [14] also reported on MDR *A. baumannii* infections in pediatric ICU patients, emphasizing the extremely high resistance rates of the isolates. In their cohort, resistance reached 100% for imipenem and extended-spectrum cephalosporins like ceftazidime, ceftriaxone, cefepime, and piperacillin–tazobactam. Resistance rates to aminoglycosides and fluoroquinolones (gentamicin, amikacin, tobramycin, ciprofloxacin) exceeded 90%. However, most isolates remained susceptible to levofloxacin, minocycline, and tigecycline. The presence of blaVIM, blaOXA-23, and blaOXA-51 genes in nearly all isolates confirmed that β-lactamases and OXA-type carbapenemases are among the predominant resistance mechanisms. Compared to these findings, the proportion of MDR strains in our cohort was notably lower, which may explain the relatively low infection-related mortality observed in our study.

Tripathi et al. [15] analyzed GNB infections in pediatric oncology patients, with leukemia being the most common diagnosis, followed by CNS tumors and bone sarcomas, a profile similar to our own cohort. Among patients with GNB infections, 70% had leukemia or lymphoma, and *Acinetobacter* spp. was the fourth most frequently identified pathogen. The mortality rate in their study was 18%. Importantly, low neutrophil counts and resistance to first-line antibiotics or combinations were significantly associated with increased mortality, whereas susceptibility to meropenem–amikacin combinations was associated with better outcomes. Interestingly, in our oncohematological disease (OHD) group, gentamicin use was associated with higher mortality, though causality remains unclear.

Finally, Talukdar et al. [16] also found *A. baumannii* to be a leading cause of infection, particularly in pediatric patients presenting with febrile neutropenia.

The primary limitation of our study lies in its retrospective design. Furthermore, since we analyzed infection episodes, rather than unique patients, we were unable to assess the potential clinical impact of recurrent infections in the same individuals. Another limitation is the absence of advanced molecular methods such as genetic sequencing, which, as demonstrated in the studies by Al-Hassan et al. [10] and Jalal et al. [11], could provide valuable insight into resistance mechanisms and strain epidemiology.

Despite these limitations, our study has several strengths. It is a multicenter, nationwide investigation covering a 12-year period and includes patients treated according to standardized, internationally approved therapeutic protocols. To our knowledge, this is the first comprehensive national study focusing on *Acinetobacter* infections among pediatric cancer patients and HSCT recipients in Poland.

## 5. Conclusions

The increasing antibiotic resistance of *Acinetobacter* spp. represents one of the most significant challenges to further progress and improved survival in pediatric oncology. In this study, we presented the incidence, clinical and microbiological characteristics, as well as treatment approaches for *Acinetobacter* infections among patients with oncohematological diseases and HSCT recipients in Poland over a 12-year period. Our findings confirm that *Acinetobacter* spp. are responsible for severe infections in this vulnerable population, with bloodstream infections being the most frequently observed clinical manifestation. This study contributes to the growing body of evidence on the public health threat posed by *Acinetobacter* spp., emphasizing the urgent need for effective infection prevention strategies and antibiotic stewardship in pediatric hemato-oncology and transplant settings.

## Figures and Tables

**Table 1:** Baseline characteristics of oncohematological diseases (OHD) group and hematopoietic stem cell transplant (HSCT) group.

Characteristics	Total	OHD Group	HSCT Group
Number of episodes	125	106	19
Sex			
Female	49	40	9
Male	76	66	10
Median age at diagnosis	5.98	5.62	1.69
Deaths			
In general	15	10	5
Treatment-related complications	4	1	3
Disease progression	7	6	1
Infection	3	2	1 (*A. baumannii* sepsis)
Unknown causes	1	1	0
Median of days from infection dg to death	86	109	72

**Table 2:** Characteristics of underlying disease in oncohematological diseases (OHD) group and hematopoietic stem cell transplantation (HSCT) group.

Diagnosis	Number of Episodes (OHD Group; *n* = 106)	Number of Episodes (HSCT Group; *n* = 19)
Hematological disorders		
Acute lymphoblastic leukemia	32	2
Acute myeloid leukemia	13	1
Lymphoma	5	2
Juvenile myelomonocytic leukemia	1	1
Myelodysplastic syndrome	0	1
Severe aplastic anemia	4	1
	Solid tumors	
Neuroblastoma	6	3
Ewing sarcoma	6	1
Central nervous system tumors	11	0
Rhabdomyosarcoma	5	0
Hepatoblastoma	4	0
Nephroblastoma	3	
Primary immunodeficiencies		
Congenital neutropenia	0	1
Chronic granulomatous disease	0	1
Other	1	1
Metabolic diseases		
Niemann Pick disease		1
Hemophagocytic lymphohistiocytosis		3
Others	15	0

**Table 3:** Microbiological and clinical characteristics of *Acinetobacter* infections in oncohematological diseases (OHD) and hematopoietic stem cell transplantation (HSCT) groups.

	OHD Group (*n* = 106)	HSCT Group (*n* = 19)
Site of infection		
bloodstream	73	16
soft tissues	10	0
gastrointestinal tract	9	1
respiratory tract	8	1
urinary tract	4	0
other	2	1
Species		
*A. baumanii*	54	13
*A. lwofii*	18	2
*A. ursingi*	9	0
*A. junii*	7	1
*A. pitii*	6	0
*A. jejuni*	1	0
*A. parvus*	1	0
*A. schindleri*	1	0
*A. johnsonii*	1	1
*A. haemolyticus*	1	0
*A. dijkshoorniae*	0	1
*A. lactucae*	0	1
*Acinetobacter* not specified	7	0

## Data Availability

The original contributions presented in this study are included in the article. Further inquiries can be directed to the corresponding authors.

## Abbreviations

The following abbreviations are used in this manuscript:

HSCT	hematopoietic stem cell transplant
OHD	oncohematological disease
ICU	intensive care unit
WHO	World Health Organization
ADC	Acinetobacter-derived cephalosporinase
ESBL	extended-spectrum beta-lactamase
ALL	acute lymphoblastic leukemia
AML	acute myeloid leukemia
CNS	central nervous system
PHO	pediatric hematology and oncology
SD	standard deviation
IQR	interquartile range
BSI	blood stream infection
RTI	respiratory tract infection
STI	soft tissue infection
UTI	urinary tract infection
MDR	multidrug-resistant
GNB	Gram-negative bacteria

## References

[1] Lee C.R., Lee J.H., Park M., Park K.S., Bae I.K., Kim Y.B., Cha C.-J., Jeong B.C., Lee S.H. (2017). Biology of *Acinetobacter baumannii*: Pathogenesis, Antibiotic Resistance Mechanisms, and Prospective Treatment Options. Front. Cell. Infect. Microbiol..

[2] Wong D., Nielsen T.B., Bonomo R.A., Pantapalangkoor P., Luna B., Spellberg B. (2016). Clinical and Pathophysiological Overview of Acinetobacter Infections: A Century of Challenges. Clin. Microbiol. Rev..

[3] DKMS Foundation 1 na 600 Dzieci Zachoruje na Nowotwór. https://www.dkms.pl/biuro-prasowe/1-na-600-dzieci-zachoruje-na-nowotwor.

[4] Ruppé É., Woerther P.L., Barbier F. (2015). Mechanisms of antimicrobial resistance in Gram-negative bacilli. Ann. Intensive Care.

[5] Lupo A., Haenni M., Madec J.Y. (2018). Antimicrobial Resistance in *Acinetobacter* spp. and *Pseudomonas* spp. *Microbiol*. Spectr..

[6] Doi Y., Murray G.L., Peleg A.Y. (2015). *Acinetobacter baumannii*: Evolution of Antimicrobial Resistance—Treatment Options. Semin. Respir. Crit. Care Med..

[7] Chávez Rodríguez M., Mascareñas De Los Santos A.H., Vaquera Aparicio D.N., Samaniego R.A., Pérez R.G., Siller-Rodríguez D., Rosales-González S.P., Castillo-Morales P.L., Bejarano J.I.C. (2024). Molecular epidemiology of carbapenemase encoding genes in *A. baumannii-calcoaceticus* complex infections in children: A systematic review. JAC-Antimicrob. Resist..

[8] Gordon N.C., Wareham D.W. (2010). Multidrug-resistant Acinetobacter baumannii: Mechanisms of virulence and resistance. Int. J. Antimicrob. Agents.

[9] Sheck E., Romanov A., Shapovalova V., Shaidullina E., Martinovich A., Ivanchik N., Mikotina A., Skleenova E., Oloviannikov V., Azizov I. (2023). Acinetobacter Non-baumannii Species: Occurrence in Infections in Hospitalized Patients, Identification, and Antibiotic Resistance. Antibiotics.

[10] Al-Hassan L., El Mehallawy H., Amyes S.G.B. (2013). Diversity in *Acinetobacter baumannii* isolates from paediatric cancer patients in Egypt. Clin. Microbiol. Infect..

[11] Jalal D., Elzayat M.G., Diab A.A., El-Shqanqery H.E., Samir O., Bakry U., Hassan R., Elanany M., Shalaby L., Sayed A.A. (2021). Deciphering Multidrug-Resistant *Acinetobacter baumannii* from a Pediatric Cancer Hospital in Egypt. mSphere.

[12] Costa P.d.O., Atta E.H., da Silva A.R.A. (2015). Infection with multidrug-resistant gram-negative bacteria in a pediatric oncology intensive care unit: Risk factors and outcomes. J. Pediatr..

[13] Shi J., Sun T., Cui Y., Wang C., Wang F., Zhou Y., Miao H., Shan Y., Zhang Y. (2020). Multidrug resistant and extensively drug resistant *Acinetobacter baumannii* hospital infection associated with high mortality: A retrospective study in the pediatric intensive care unit. BMC Infect. Dis..

[14] Zhu Y., Zhang X., Wang Y., Tao Y., Shao X., Li Y., Li W. (2022). Insight into carbapenem resistance and virulence of Acinetobacter baumannii from a children’s medical centre in eastern China. Ann. Clin. Microbiol. Antimicrob..

[15] Tripathi R., Jain P., Tarai B., Arora R. (2023). Factors associated with mortality from gram-negative bacterial infections in children with cancer. Pediatr. Hematol. Oncol. J..

[16] Talukdar A., Barman R., Hazarika M., Das G. (2023). Bloodstream Infections in Pediatric Cancer Patients with Febrile Neutropenia at a Tertiary Cancer Center in Northeast India. Asian Pac. J. Cancer Care.

